# Immunophenotype of lymphocytes and real-world outcome of COVID-19 infection in children with hematology and oncology

**DOI:** 10.1186/s12885-024-12262-1

**Published:** 2024-04-27

**Authors:** Na Zhang, Zhen Wang, Hong Li, Kai Chen, Hong-sheng Wang, Jing-bo Shao, Sha-yi Jiang, Xiao-wen Zhai, Hui Jiang

**Affiliations:** 1 Department of Hematology and Oncology, Shanghai Children’s Hospital, School of medicine, Shanghai Jiao Tong University, 200040 Shanghai, China; 2 Department of Hematology and Oncology, Children’s Hospital of Fudan University, Shanghai, China

**Keywords:** COVID-19, Children, Immunocompromise, Immunophenotype

## Abstract

**Background:**

Patients with immunocompromise were suspected to encounter a high risk for severe coronavirus disease 2019 (COVID-19) infection on early period; however, data is lacking nowadays and immune response remain unclear.

**Methods:**

In this retrospective study, internet questionnaire survey and medical records were acquired in pediatric hematology oncology patients. Clinical severity, immunological characteristics, and outcomes were analyzed from December 1, 2022 to January 31, 2023 at the 3rd year of pandemic in China.

**Results:**

A total of 306 patients were included, with 21 patients (6.9%) asymptomatic, 262 (85.6%) mild severity, 17 (5.6%) moderate severity, 5 (1.6%) severe severity, and 1 (0.3%) critical severity. Seventy-eight (25.5%) patients were on intensive chemotherapy, and 32.0% children were on maintenance chemotherapy. Delays in cancer therapy occurred in 86.7% patients. Univariable analysis revealed active chemotherapy (*P* < 0.0001), long duration of symptom (*P* < 0.0001), low lymphocytes count (*P* = 0.095), low CD3 + and CD8 + T cell count (*P* = 0.013, *P* = 0.022), high percentage of CD4 + TCM (*P* = 0.016), and low percentage of transitional B cells (*P* = 0.045) were high risk factors for severe COVID-19 infection. Cox regression model showed that the absolute lymphocytes count (*P* = 0.027) and long duration of symptom (*P* = 0.002) were the independent factors for severity. Patients with CD8 + dominant and B cell depletion subtype wasn’t related with severity, but had higher percentage of CD8 + effector memory T cells (TEM) and terminally differentiated effector memory T cells (TEMRA) (*P* < 0.001, *P* < 0.001), and a longer COVID-19 duration (*P* = 0.045).

**Conclusion:**

The severity was relatively mild in children with immunodeficiencies in the third year of COVID-19 pandemic. Low lymphocyte count and long duration of symptom were the independent risk factors with COVID-19 severity. Delays in cancer care remain a major concern and the long outcome is pending.

**Supplementary Information:**

The online version contains supplementary material available at 10.1186/s12885-024-12262-1.

## Introduction

The coronavirus disease 2019 (COVID-19) has caused a global epidemic of severe acute respiratory syndrome coronavirus 2 (SARS-CoV-2) since December 2019. Since February 2022, Omicron, as the fifth variant, showed the dominant subtype in China. Starting from December 2022, there has been a peak of infection in the population, as well as pediatric patients with hematology and oncology disease.

Comparing adult population, the pediatric population showed a milder severity of clinical course and a lower rate of mortality [1, 2]. Approximately 90% patients expressed an asymptomatic, mild, or moderate severity in children [2–4]. Compared with the general population, most patients with hematology and oncology diseases were immunocompromised and may be at a high risk of infection. Early reports from developing and developed country, showed a higher proportion of severe disease in adult patients with oncology disease [5, 6]. Children undergoing anticancer therapy or immunotherapy have been supposed to suffer with severe COVID-19 as well. In children with cancer, the rate of severe infection ranged from 10 to 20.1% and mortality ranged from 1.6 to 4.9% in 2020–2021 reports [7–10]. There may be changes due to the variance of Omicron as the dominant prevalent variety nowadays. However, data to support this is lacking, especially in children with immunodeficiencies.

Recent studies suggest that T lymphocytes play a significant role in the pathological process of COVID-19, particularly in CD4 + T and CD8 + T cells [11, 12]. Additionally, Natural killer (NK) cells are an original protection against viruses [13]. Abnormal T cells or NK cells in immunocompromised patients may reduce the capacity the virus clearance. The knowledge for T cells phenotype accompanied COVID-19 remains insufficient, especially in immunocompromised children. In this study, we identified the clinical characteristics in this highly vulnerable immune-compromised population. We analyzed CD4 + and CD8 + T cell of COVID-19 infected cases between basic immune status and recovery, together with the phenotypic status of T cells. Our findings might provide insights for understanding T cell immunity for controlling COVID-19 in immunodeficiencies.

## Methods

### Patients

Pediatric patients with COVID-19 infection were enrolled in this two-center, retrospective research from December 1, 2022 to January 31, 2023. Inclusion criteria meet all the followings: (1) age ≤ 18 years; (2) COVID-19 infection diagnosed by nasopharyngeal swab using rapid antigen test or reverse transcription polymerase chain reaction; (3) patients have ever undergone chemotherapy, immunosuppressive therapy, immunotherapy, radiotherapy, or have received stem cell transplantation (SCT); (4) patients or their parents finished the internet questionnaire survey. Patients missing important information have been excluded.

### Study design

The questionnaire (Supplementary Material 1) was used including demographic, underlying disease, the dates of COVID-19 test showing positive and negative, clinical symptoms, COVID-19 directed treatment, hospital stay, vaccination of COVID-19, and consequences for disease. Data of the treatment was acquired in the medical record system. Immune parameters were assessed before COVID-19 diagnosis and at resolution of the disease. The underlying disease therapy was suggested to be postponed for 1–2 weeks until recovery from COVID-19. COVID-19 recovery was defined as clearance of clinical symptoms or nasopharyngeal swab test negative from initial diagnosis in asymptomatic patients. The study was performed under the Declaration of Helsinki and approved by the local Ethics Committee (No. 2023R027-E01). Patients’ follow-up was updated on February 28, 2023.

### Disease severity definitions

Clinical severity was classified according to the New Coronavirus Pneumonia Prevention and Control Program (9th edition). Asymptomatic: no symptoms at any time point; Mild: fever and or respiratory symptoms, without pneumonia; Moderate, pneumonia with fever or respiratory tract symptoms; Severe, respiratory rate ≥ 30/min, oxygen saturation ≤ 93%, or PaO2/FiO2 ≤ 300 mmHg; Critical, respiratory failure demanding mechanical ventilation, shock, or organ failure requiring intensive care.

### Chemotherapy intensity definitions

Intensive chemotherapy included induction and consideration in acute leukemia, lymphoma, and solid tumor. Maintenance therapy included oral and low-medium intensive chemotherapy in remission or immunosuppressive therapy for benign hematologic disorder. Blinatumomab, rituximab or chimeric antigen receptor (CAR) T cell (days more than 30 days from CAR infusion) targeted immunotherapy was grouped into maintenance therapy. Transplantation within 3 months was grouped into intensive chemotherapy status. Transplantation away from 3 months or receiving management of graft-versus-host disease was grouped into maintenance therapy.

### Lymphocyte subtype by flow cytometry

The basic and fine lymphocyte subpopulations were performed using a FACSCalibur flow cytometer (BD Biosciences) and reported as the percentage and the absolute counts. Flow cytometry (FCM) was performed for the analysis of basic lymphocyte subsets (CD3/CD45/CD4/CD8/CD16CD56, BD Biosciences) before COVID-19 diagnosis and at recovery, including T cells (CD3 + CD45+), cytotoxic T cells (CD3 + CD8 + CD45+), helper T cells (CD3 + CD4 + CD45+), NK cells (CD16 + CD56 + CD3 − CD45+), and B cells (CD19 + CD45+). The 15,000 lymphocytes were acquired for analysis. In addition, subtle lymphocyte type was performed and the antibodies were purchased from BD Biosciences. Naive T cells (CD45RA + CD27+), central memory T cells (TCM) expressed with CD45RA − CD27+, effector memory T cells (TEM) with CD45RA − CD27−, and terminally differentiated effector memory T cells (TEMRA) with CD45RA + CD27 − were examined. Meantime, naïve B (CD19 + CD27 − IgD+), memory B (CD19 + CD27 + IgD−), transitional B (CD19 + CD24 + + CD38++), plasmablast (CD19 + CD24 − CD38++) were calculated. The 30,000 to 50,000 lymphocytes were acquired for the analysis. B cell depletion defined as proportion less than 3%.

### Statistical analysis

The quantitative data with Gaussian distribution were presented as the mean ± standard deviation, using t-test comparison. Non-normally distributed data were presented as median and full range. Data was analyzed by the Mann-Whitney test or Wilcoxon matched pairs test for two groups, and one-way analysis of variance and Kruskal-Wallis test among 3 or more groups. Categorical variables were present with percentage, using Fisher’s exact test or chi square (χ^2^) test. Statistical analyses were performed with SPSS 19.0 and GraphPad Prism 9.5 at a significance level of 0.05 of two-sided.

## Results

### Patients’ characteristic

A total of 306 patients were enrolled. Patients’ age ranged from 8 months to 18 years old. Nearly half of the patients (176, 57.5%) were on underlying disease therapy, including intensive chemotherapy (25.5%) and maintenance chemotherapy (32.0%). Eight patients received immunotherapy including blinatumomab, rituximab or CD19/CD22-directed CAR T therapy. In 52 patients who underwent transplantation, 23 patients were receiving immunosuppressive therapy. There was no patient actively undergoing allogeneic SCT within 1 month. The total demographic data of COVID-19 patients were showed in Table 1.

**Table 1:** Clinical characteristics for COVID-19 infection in patients with hematology and oncology disease

Characteristics	Total (%) *n* = 306	Asymptomatic /Mild *n* = 283	Moderate/Severe *n* = 23	Univariable Analysis *P* value
Age, years				0.668
< 1	12 (3.9)	11	1	
1–10	145 (47.4)	133	12	
≥10	149 (48.7)	139	10	
Gender				0.126
Male	181 (59.2)	171	10	
Female	125 (40.8)	112	13	
Diseases				0.440
Malignant hematology^*^	193 (63.1)	175	18	
Lymphoma	24 (7.8)	24	0	
Solid tumor	8 (2.6)	8	0	
Benign hematologic disorder ^†^	29 (9.5)	27	2	
Post SCT	52 (17.0)	49	3	
Underlying disease course				< 0.0001
Intensive chemotherapy	78 (25.5)	62	16	
Maintenance chemotherapy	98 (32.0)	95	3	
Stopped chemotherapy	130 (42.5)	126	4	
<1 year from stopping	28 (9.2)	-	-	
1–3 years from stopping	54 (17.6)	-	-	
≥3 years from stopping	48 (15.7)	-	-	
Target B cells immunotherapy^#^				0.115
Yes	8 (2.6)	6	2	
No	298 (97.4)	277	21	
Vaccination^$^				0.805
Yes (any dose)	70 (25.8)	64	6	
No	201 (74.2)	185	16	
In immune-compromise stage				0.793
Vaccinated	47 (25.8)	41	6	
Non-vaccinated	135 (74.2)	120	15	
COVID-19 continuing time, days (range)	7 (1–35)	7 (1–23)	6.5 (4–35)	0.753
Duration of symptom, days (range)	4.0 (1.0–20)	3.5 (1.0–20)	7.0 (2.0–18)	< 0.0001

^*^ Including Acute lymphoblast leukemia, Acute myeloid leukemia, Chronic myeloid leukemia; ^†^ Including Langerhans cell histiocytosis, Immune thrombocytopenia, and Aplastic anemia receiving immunosuppressive therapy; ^#^ Including CD19 or CD22-directed CAR T therapy, rituximab, or blinatumomab therapy; ^$^ 35 cases with no answer to the questionnaire. *SCT*, stem cell transplantation

### Severity of COVID-19 diseases

**Fig. 1:**
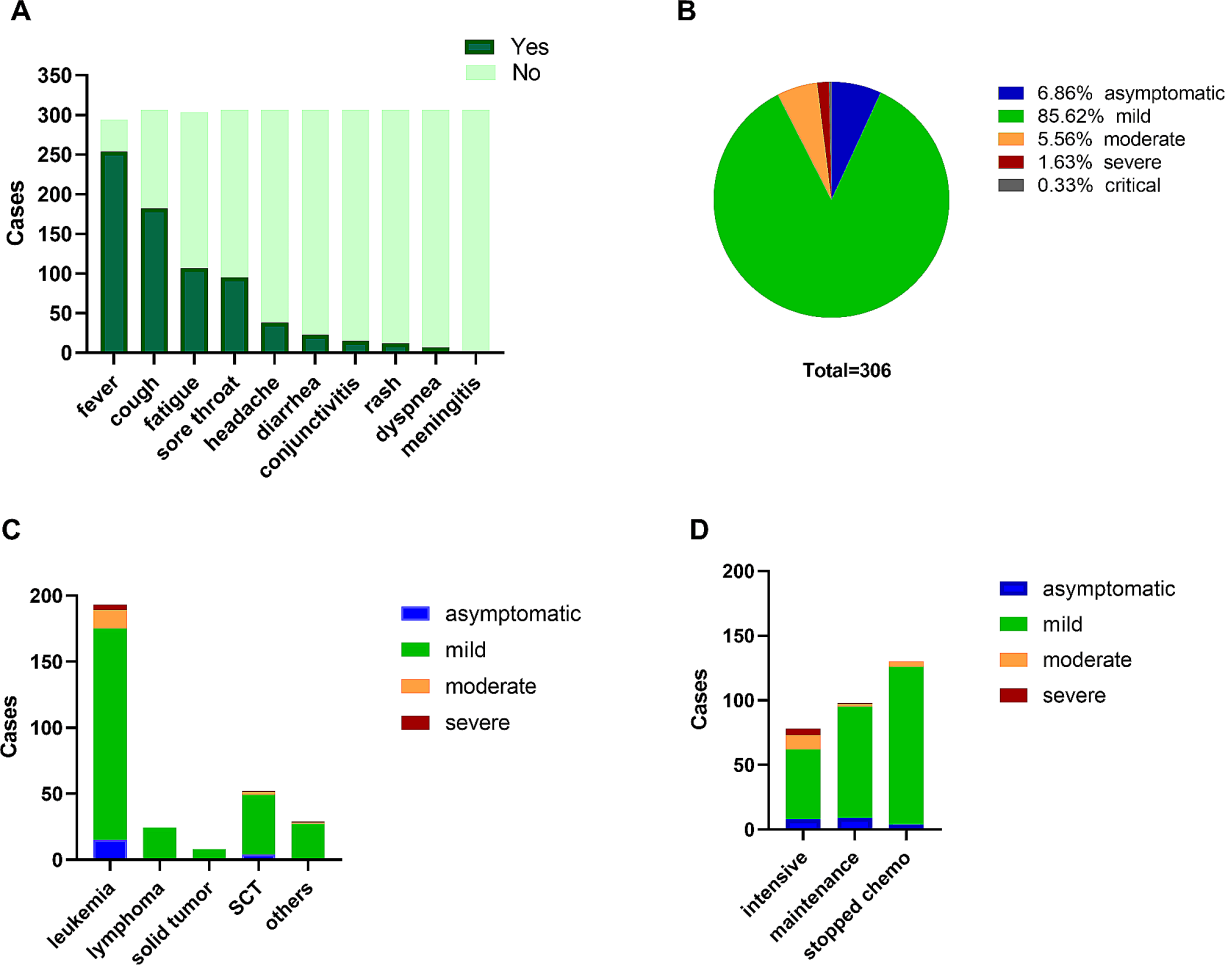
Symptoms and disease severity of patients with COVID-19 infection. **A**: symptoms of COVID-19; **B**: severity of COVID-19 infection in whole cohort; **C**: COVID-19 severity of underlying disease type; **D**: COVID-19 severity of underlying disease course

Severity was asymptomatic in 21 children (6.9%), mild in 262 (85.6%), and moderate in 17 (5.6%). 6 patients were reported as serious COVID-19 infection, severe (*n* = 5, 1.6%) and critical infections (*n* = 1, 0.3%) (Fig. 1B). There was no difference in the severity distribution of COVID-19 infection among different types of hematological and oncological diseases (Fig. 1C; Table 1). In 176 patients under chemotherapy, severe and critical severity was reported in 6 cases (3.4%) and 1 case died (0.6%). Twenty-five patients required admission to the hospital; and 1 child required mechanical ventilation and transferred to the intensive care unit. The most commonly presenting symptoms included fever (86.4%), cough (59.4%), fatigue (35.3%), and sore throat (31.0%) with vomiting/diarrhea, headache, and conjunctivitis being reported less frequently (Fig. 1A). The fever lasted from 1 day to 11 days with the highest temperature to 42℃. Other rare symptoms included rash, myalgias, and anorexia.

### Risk factors of COVID-19 diseases

The data of lymphocyte subsets was available in 109 patients who recovered from COVID-19. Eighty-three (76.1%) patients were in underlying disease treatment stage, 12 patients (11.0%) have stopped therapy within one year, and 14 patients (12.8%) have stopped therapy more than one year. The possible risk factors of severity of COVID-19 infection were shown in Table 2. Univariable analysis revealed that low absolute CD3 + T cell count (*P* = 0.013), low CD8 + T cell count (*P* = 0.022), high percentage of CD4 + TCM cells (*P* = 0.016), and low percentage of transitional B cells (*P* = 0.045) were the risks for severe severity (Table 2). The absolute lymphocytes counts were variable and showed a mild relation to clinical severity (*P* = 0.095). Severity of COVID-19 were not associated with the absolute B cell counts and the subtype of T cells (*P* = 0.436, 0.893; Table 2). We also found active chemotherapy (*P* < 0.0001, Fig. 1D; Table 1) and long duration of symptom (*P*<0.0001; Table 1) were associated with the severity. Further Cox regression model analysis showed that the absolute lymphocytes count (*P* = 0.027) and long duration of symptom (*P* = 0.002) were independent prognostic factors for severity.

**Table 2:** Risk factors of immune features for COVID-19 infection in patients with immune-compromise

Features	Asymptomatic/Mildn=95	Moderate/Severen=14	Univariable analysis P value
Lymphocyte, /μL	1364 (284-6382)	1084 (274-3107)	0.095
CD3+ T cell, /μL	1167 (254-6261)	706.5 (104-2113)	0.013
CD8+ T cell, /μL	628 (49-3913)	336 (51-1066)	0.022
CD4+ T cell, /μL	354 (60-3554)	307 (18-881)	0.108
CD19+ B cell, /μL	26 (0-1719)	24.5 (0-742)	0.436
NK cell, /μL	160 (3-1477)	95.5 (7-1869)	0.909
CD4+/CD8+ ratio	0.7 (0.1-2.8)	0.8 (0.3-2.3)	0.540
T and B cell subsets			0.893
CD8^dom^ B^dep^	33	5	
CD8^dom^ B^+^	32	3	
CD4/CD8^nor^ B^dep^	16	3	
CD4/CD8^nor^ B^+^	10	2	
CD4^dom^ B^+^	4	1	
CD8+ T cells			
Naïve, %	48.4 (6.1-97.2)	57.2 (1.7-86.1)	0.993
TCM, %	16.3 (1.6-59.2)	24.1 (8.0-63.7)	0.194
TEM, %	5.7 (0.2-55.8)	8.7 (1.2-32.1)	0.685
TEMRA, %	13.3 (0.3-70.8)	9.0 (1.0-33.7)	0.404
CD4+ T cells			
Naïve, %	41.3 (2.6-92.5)	41.4 (1.0-70.6)	0.338
TCM, %	37.4 (5.4-65.9)	41.8 (22.2-89.3)	0.016
TEM, %	12.6 (0.3-65.2)	11.9 (4.6-45.7)	0.685
TEMRA, %	1.1 (0-15.0)	1.0 (0-3.7)	0.525
B cells			
Naïve B, %	65.0 (0-97.1)	24.1 (0-97.8)	0.095
Memory B, %	8.6 (0- 68.8)	11.6 (0-61.0)	0.807
Plasmablast, %	1.6 (0-50.0)	5.6 (0-35.6)	0.864
Transitional B, %	9.8 (0-76.7)	0.8 (0-69.2)	0.045

*NK cells*, Natural killer cells; *TCM,* central memory T cells; *TEM*, effector memory T cells; *TEMRA*, terminally differentiated effector memory T cells; *SCT*, stem cell transplantation; *dom*, dominant; *dep*, depletion; *nor,* normal.

### Immune response post COVID-19 infection

**Fig. 2:**
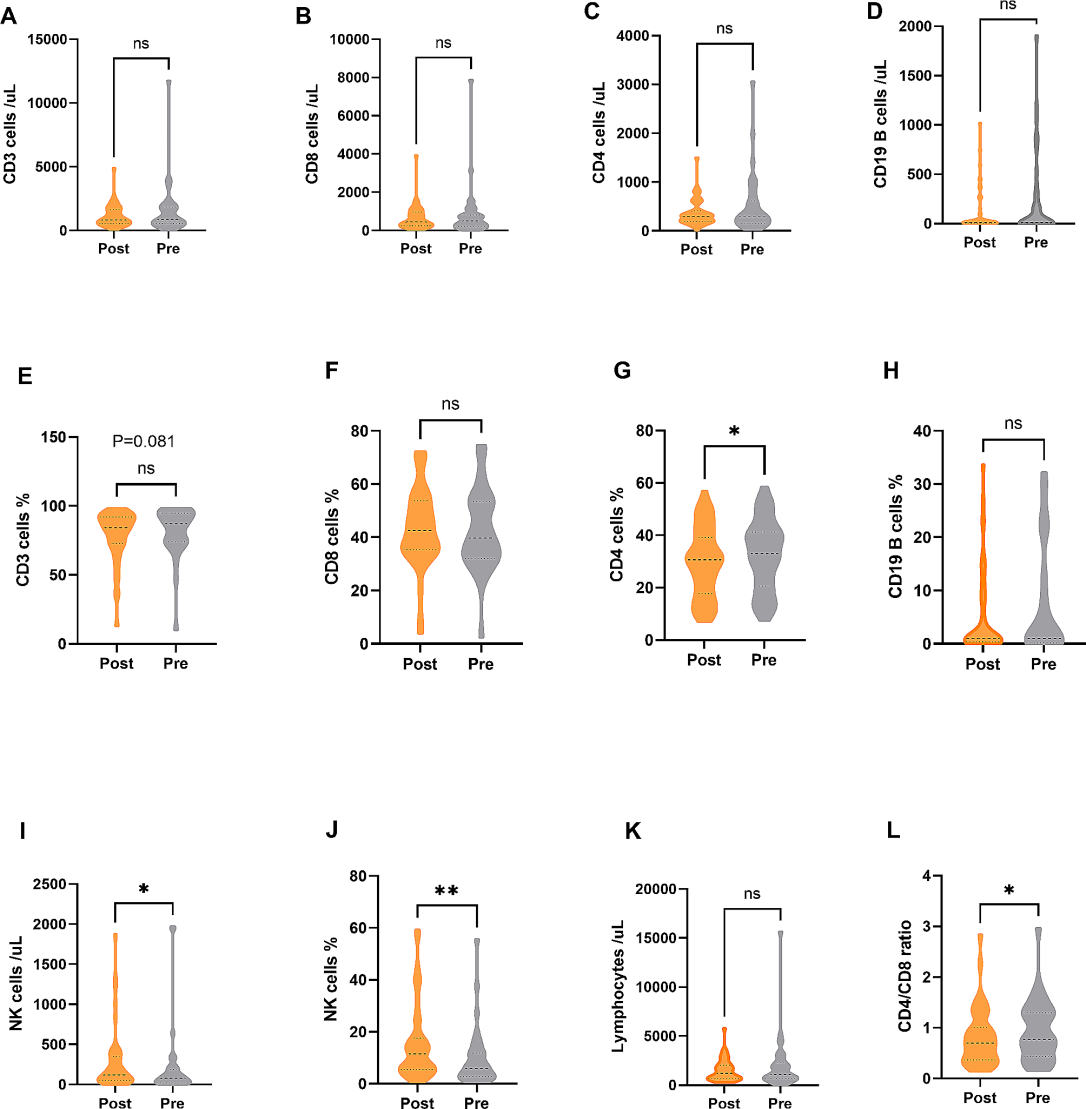
Lymphocyte subtype in patients with hematological and oncological disease post COVID-19 infection compared with basic status. **A-D**: The absolute levels of CD3+, CD4+, CD8 + T cells, and CD19 + B cells; **E-H**: The frequency of CD3+, CD4+, CD8 + T cells, and CD19 + B cells; **I-J**: absolute and frequency of NK cells; **K**: absolute lymphocyte counts; **L**: CD4+/CD8 + ratio. *P < 0.05, * *P < 0.01 (Pair t test or Wilcoxon matched-pairs signed rank test)

Laboratory parameters of lymphocyte subtype were collected before COVID-19 diagnosis and at the resolution of the disease within 3 weeks after diagnosis. The absolute counts of CD3 + and CD8 + T cells were lower in recovery status compared with primary status, and CD4 + and B cells fluctuated mildly, but all with no significance (Fig. 2A-D). While NK cells were noticed significantly increasing during the infection (*P* = 0.026, Fig. 2I). Furthermore, CD4 + T cells proportion showed a lower frequency in recovery status than primary status (29.6% vs. 32.5%, *P* = 0.049, Fig. 2G). But mildly decreased CD3 + T proportion was seen in recovery status (*P* = 0.081, Fig. 2E). The proportion of CD8 + T and B cells fluctuated mildly during the infection (*P* = 0.329, 0.377; Fig. 2F, H). A lower CD4+/CD8 + ratio was seen in recovery patients than primary status (0.70 vs. 0.77, *P* = 0.021, Fig. 2L). NK cells proportion was higher in recovery status than primary status (11.6% vs. 6.0%, *P* = 0.002, Fig. 2J). The total lymphocyte was similar during the infection (*P* = 0.768, Fig. 2K).

**Fig. 3:**
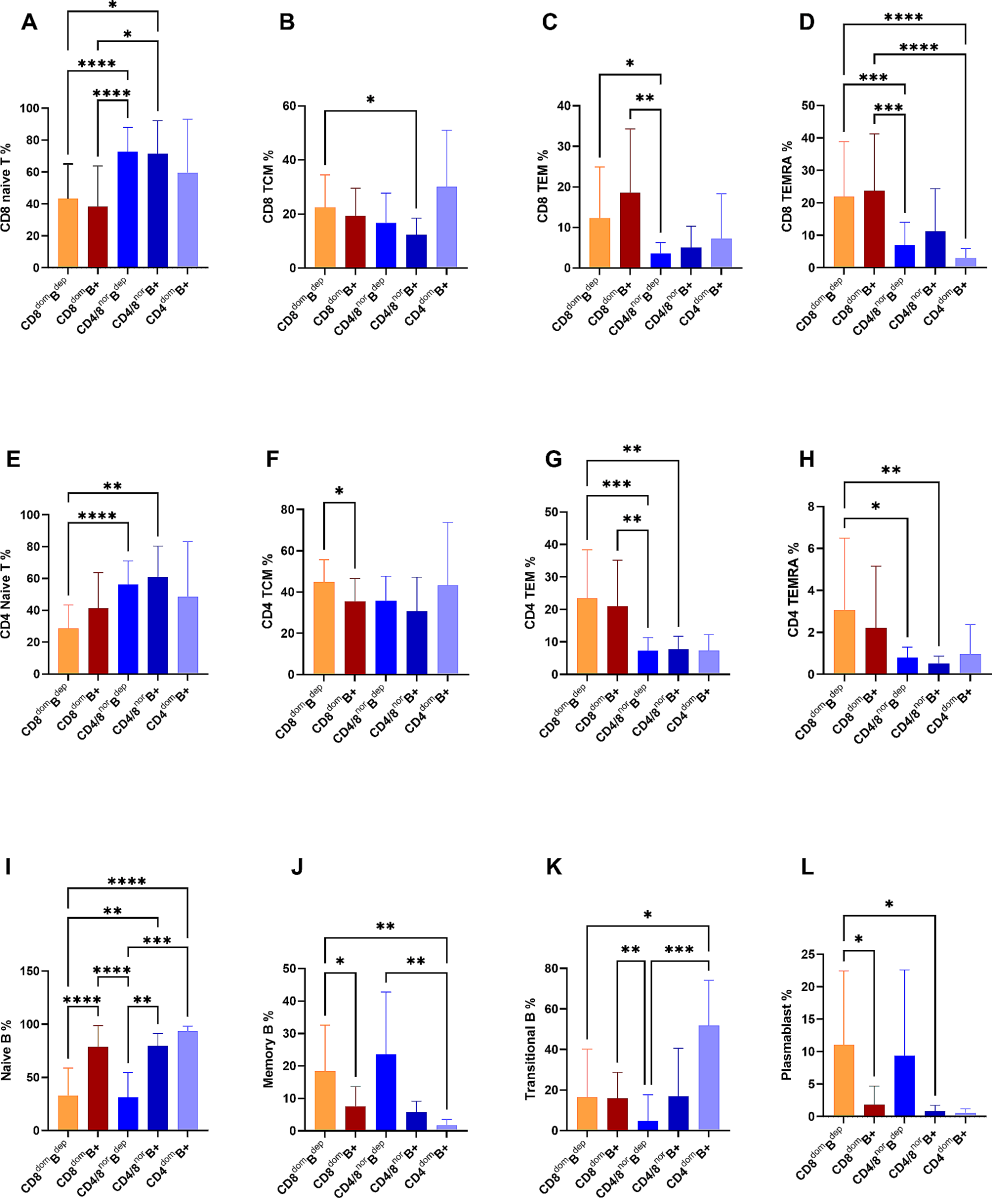
T cell exhaustion and B cell response in COVID-19 patients with defined phenotype. **A-D**: CD8 + T cell differentiation of Naïve T, TCM, TEM, and TEMRA in 5 groups with CD8^dom^B^dep^, CD8^dom^B^+^, CD4^dom^B^+^, CD4/CD8^nor^B^dep^, and CD4/CD8^nor^B^+^; **E-H**: CD4 + T cell differentiation of Naïve T, TCM, TEM, and TEMRA in 5 groups; **I-L**: B cell response of Naïve B, memory B, transitional B and plasmablast in 5 phenotypic groups. *P < 0.05, * *P < 0.01, ***P < 0.001 (Kruskal-Wallis test or Brown-Forsythe ANOVA test)

According to the subtle subtype of T and B cells at resolution of COVID-19 infection, we further divided patients by CD8 + dominant and B cell depletion (CD8^dom^B^dep^, 35.8%), CD8 + dominant and B cell positive (CD8^dom^B^+^, 31.2%), B cell positive and a CD4-dominant (CD4^dom^B^+^, 4.6%), normal CD4+/CD8 + T cell ratio and B cell depletion (CD4/CD8^nor^B^dep^, 17.4%), or balanced CD4+/CD8 + and B cells positive (CD4/CD8^nor^B^+^, 11.0%). The data of subtle subtypes were available in 90 patients. Comparison with groups, patients with CD8^dom^B^dep^ had very lower frequency of naive T cells but higher frequency of CD8^+^ TEM and TEMRA cells than CD4/CD8^nor^B^dep^ (Fig. 3A, C,D). TCM CD8 + cells expressed higher in CD8^dom^B^dep^ group than CD4/CD8^nor^B^+^ (Fig. 3B). CD8^dom^B^dep^ had lower frequency of CD4^+^ naive T cells but higher frequency of CD4^+^ TEM and TEMRA cells comparing with CD4/CD8^nor^B^dep/+^ (Fig. 3E, G, H). CD8^dom^B^dep^ had lower frequency of naïve B cells than CD8^dom^B^+^, CD4/CD8^nor^B^+^ and CD4^dom^B^+^; but had higher frequency of memory B cells than CD8^dom^B^+^ and CD4^dom^B^+^. Transitional B cells expressed lower in CD8^dom^B^dep^ than in CD4^dom^B^+^. CD8^dom^B^dep^ had very higher proportions of plasmablast than CD4/CD8^nor^B^+^ and CD8^dom^B^+^ (Fig. 3I-L). Other expression of T and B cells phenotypes were showed in Fig. 3.

### Treatment of COVID-19 and delay of underlying disease treatment

Most patients (85.6%) were in quarantine at home with antipyretic or antitussive therapy. 6 (2.0%) patients need supplemental oxygen requirement. Twenty-one (6.9%) patients received anti-virus therapy; intravenous gamma globulin (84.0%) and oseltamir (40.0%) were used frequently in inpatients. Paxlovid was used in 1 severe patient. Remdesivir or convalescent plasma were not used in this cohort. Reported complications from COVID-19 included bacterial or fungus superinfection in 7.5%, respiratory failure in 2.0%, and encephalitis in 0.7% of all cases. *Klebsiella pneumoniae, stenotrophomonas maltophilia, or enterococcus faecalis* has been detected from phlegm, bronchoalveolar lavage fluid or blood. Two cases were coinfected with *mycoplasma*. Patients combined with co-infection had a risk for severe disease (65.2% vs. 2.8%, *P* < 0.0001). Those patients usually under intensive chemotherapy with myelosuppression. Overall, one death was reported in the cohort with relapsed acute lymphoblastic leukemia in reinduction phase. The patient developed *Klebsiella pneumoniae* sepsis during the period of COVID-19 infection. Of the 158 patients who under anticancer therapy, there were interruptions in 86.7% of patients, with a median of 10 days delay ranging from 5 to 21 days. Reasons for delays included high fever, underlying myelosuppression, at myelosuppressive phase, or other complication.

Median time to symptom disappearance of COVID-19 was 4 days (range 1–18 days). Median time for COVID-19 clearance was 7 days (range 1–35). Patients with CD8^dom^B^dep^ subtype had a longer COVID-19 duration of 9.1±4.0 days, compared with 6.8±2.8 days in CD8^dom^B^+^ (*P* = 0.045), 6.0±1.5 days in CD4/CD8^nor^B^dep^ (*P* = 0.001), and 4.8±2.3 days in CD4/CD8^nor^B^+^ (*P* = 0.001). Only 5 patients were included in CD4^dom^B^+^ subset and had a COVID-19 duration time of 13±12.9 days. Patients with CD4/CD8^nor^B^+^ had the quickest clearance of COVID-19, but there was no deference with patients with CD8^dom^B^+^, CD4/CD8^nor^B^dep^, or CD4^dom^B^+^ subset. The clearance day (≤ 7 days) was not related with B cell level (*P* = 0.876), CD8 + level (*P* = 0.803), CD3 + value (*P* = 0.873), and severity (*P* = 0.367).

## Discussion

We analyzed patients who suffered COVID-19 infections in pediatric hematology oncology department in late 2022 to early 2023, suggesting a favorable prognosis of COVID-19 infection. In our cohort, the majority patients presented asymptomatic to mild symptoms, with a low morbidity of 0.3%. The only death with SARS-CoV-2 infection accompanied with sepsis. Reports had variance among groups and population. Severe rate was reported higher in pediatric patients with cancer than general population in early period of COVID-19 epidemic [7–10]. In contrast, reports in Italy showed that there were no serious or critical cases of COVID-19 in children with cancer, which was similar to general pediatric population [14]. Similar result was seen in the days of the emergence of new virus variants when the epidemiology of the pandemic changed [15].

Cellular immunity takes part in controlling viral infections. The exhausted phenotype with decreased counts of CD4 + T cells and CD8 + T cells were seen in severe course of COVID-19, resulting a reduced CTL functionality [16]. Moreover, lymphopenia was commonly observed in acute phase of COVID-19, particularly in severe disease [17], suggesting T cell function impairment and apoptosis. In our cohort, patients were usually at low number of CD4+, CD8+, and B cells before COVID-19 infection. We observed the decreasing percentage of CD3 + and CD4+, and CD4+/CD8 + ratio even at recovery period. However, Mahmoudi S et al. found that lymphocytopenia rarely occurred in general children with mild infection of SARS-CoV-2, while lymphopenia mostly appeared in severe disease [11]. Kuczborska K et al. found children have no tendency to lymphopenia, regardless of the severity of COVID-19 [12].

CD8 + cytotoxic lymphocytes are responsible for interrupting viral replication and killing infected cells, which is negative with the disease progression [16]. CD4 + T cells are the key pieces of regulating antiviral immunity and may be related with severity [18]. CD4 + T cells showed mildly low in moderate/severe patients, while lymphocyte counts, CD3 + T cells, and CD8 + T cells counts showed significantly lower in our cohort. That was consistent with previous research [19]. Furthermore, the count of lymphocyte was the independent factor for severity risk. The values of specific lymphocyte subsets are controversial. Several researches present higher ratio of CD4+/CD8 + T cell with more severity and greater lung involvement in both general [20] and immunocompromised children [12], suggesting decreased CD4+/CD8 + T cell ratios in children with immunocompromise may be a protective factor for the prevention for severe COVID-19. However, opposite opinions have reported higher CD8 + T cell level [21] and lower CD4+/CD8 + T cell ratio [11, 21] have been seen in children with pneumonia. CD4+/CD8 + ratio showed no relation with severity in our group, suggesting a complementary effect of other immune cells in the process of infection. In addition, study population differed from the studies should be noted, which may substantially impact obtained results. Also, recipients of receiving intensive chemotherapy presented a higher severity, which was similar to other reports [15, 17]. These population usually had very low number of lymphocytes and neutrophils, indicating a high risk of coinfection.

NK cells are innate lymphocytes for infection. NK decreasing in absolute number have been observed alongside lymphopenia in healthy population epidemic [22, 23], but the proportion of NK cells stayed in normal range. However, few study was showed no difference between the healthy donors, mild severity, and severe severity; either in the proportion of NK cells or absolute number [24]. In our study, NK cells count was basically low for immunodeficiency. Percentage of NK cells ranged widely due to chemotherapy or immunosuppression. NK cells have been observed with increased number and frequency of in our immunocompromised group at recovery for the probable reasons of withdraw of immunosuppression.

Naïve T cells participated in primary infection response and the subsequent antigen specific immune responses induced by memory T cells. T cells with TEMRA phenotype was the sigh of exhaustion. We further found that there was no difference of CD8 + Naïve T, TCM and TEMRA in asymptomatic/mild and moderate/severe disease. CD4 + TCM showed a higher percentage in group of high severity but with no risk in Cox regression model analysis. The other author observed a bias differentiation towards TCM in mild disease and TEMRA in severe severity [25, 26]. We didn’t find this bias in recovery period possibly due to the small samples of severe cases. We found CD8^dom^B^dep^ subtype had very low frequency of naive T cells but high proportions of TEMRA CD8^+^ T cells as found in study [27]; similar differentiation was showed in CD4 + T cells. Interesting, CD8^dom^B^dep/+^ had longer duration of COVID-19, which is the most commonly immune phenotype of immunodeficiencies in hematology oncology patients.

Low humoral immunity showed a possible risk of COVID-19 clearance in hematologic malignancies patients in early reports. CD19 + B cell was generally low in patients with hematology and oncology diseases in this cohort. However, most patients recovered from COVID-19 ultimately, including those who had received therapies for B cell clearing. While in groups with B cell depleted or positive, there was no difference in severity, and viral clearance days. In addition, we found low percentage of transitional B cells in high severity, which may be related with immunosenescence. Viral duration time was not related with the absolute B cell as in paper reported [27], and CD8 + T cells. Moreover, CD8 + T cell seemed to compensate for the lack of humoral immunity in haematological malignancy patients, and was related to improved prognosis [28].

Another concern was the high rate for delays in chemotherapy that accompanied COVID-19. In the early pandemic phase, cancer-directed therapy was usually postponed with presuming a risk for severe COVID-19 due to their immunocompromise [12]. For most reports, the children with malignant diseases mostly showed non-severe clinical courses of COVID-19 infection. But compared to hospitalized children without comorbidities, the mortality is at least 10 times higher [29]. In several cohort studies, chemotherapy was continued despite COVID-19 positivity with individual chemotherapy modification without severe COVID-19 complications [29], or regular protocol continuation on maintenance therapy [15]. We conducted the short time withdrawal of therapy, and the influence to prognosis was not sure. For patients with high severity, duration of symptom was longer in our report. That may delay more time for underlying disease treatment. The further study need perform to determine the potential impact of the pandemic on long time survival.

## Conclusions

We reported the outcome of COVID-19 infection in children with hematology and oncology diseases in this big pandemic wave in China, presenting mainly asymptomatic or mild symptoms. High lymphocytes, CD8 + T cells or CD3 + T cells may be helpful for controlling infected cells and seem to compensate humoral immunodeficiency in hematological and oncological patient. Decreased CD4+/CD8 + ratio and increased NK cells were seen after infection. CD8^dom^B^dep^ subtype usually expressed an exhausted phenotype in long COVID-19 continuation, but not differ in severity.

### Electronic supplementary material

Below is the link to the electronic supplementary material.

Supplementary Material 1

## Data Availability

Data supporting this research findings were collected from registries and medical records at Hematology and Oncology Department, Shanghai Children’s hospital. The datasets generated and/or analyzed during the current study are available from the corresponding author on reasonable request.

## Abbreviations

COVID-19: Coronavirus disease-2019
CAR T: Cell chimeric antigen receptor T cell
NK cells: Natural killer cells
SCT: Stem cell transplantation
SARS-CoV-2: Severe acute respiratory syndrome coronavirus-2
TCM: Central memory T cells
TEM: Effector memory T cells
TEMRA: Terminally differentiated effector memory T cells
