# Supplementary material for: Immunophenotype of lymphocytes and real-world outcome of COVID-19 infection in children with hematology and oncology
Source: BMC Cancer. 2024 Apr 27;24:538. doi: 10.1186/s12885-024-12262-1 (PMC11056048; doi:10.1186/s12885-024-12262-1)
Supplement: Supplementary file 1 — Supplementary Material 1 [file 12885_2024_12262_MOESM1_ESM.docx]

**Questionnaire of COVID-19 infection in Hematology and Oncology department**

Question 1: Child’s name: .

Question 2: Child’s primary disease [multiple choices]

Tick in the options

Acute leukemia

Chronic leukemia

Lymphoma

Receiving transplantation

Langerhans cell histiocytosis

Aplastic anemia

Neuroblastoma

Nephroblastoma

Hepatoblastoma

Medulloblastoma

Retinoblastoma

Other blood disease

Question 3: Family members of COVID-19 infection and the date (e.g. March 4, 2022) [multiple choices]

Farther, date: .

Mother, date: .

Child, date: .

Other members, date: .

Question 4: Suspected infection route of the child's COVID-19 infection [multiple choice]

Nucleic acid detection station

In hospital

Leaving residence

Others

Question 5: Suspected infection route of the family members [multiple choice]

Nucleic acid detection station

In hospital

Leaving residence

Others

Question 6: Has the child been to the outpatient clinic or been hospitalized one week before COVID-19 infection? Please fill the dates (e.g. March 4, 2022) [multiple choices]

Visiting outpatient clinic (including fever clinic), date: .

Hospitalization, date: and length of stay: days

None of the above

Question 7: What kind of transportation was used for visiting the hospital one week before infection [multiple choice]

Aircraft

Train

Coach

Driving a car

Patient living in Shanghai

Not visiting hospital before infection

Question 8: Detection method of COVID-19 [single choice]

Nucleic acid

Antigen

Nucleic acid + antigen

Question 9: The symptoms of COVID-19 infection of the child [multiple choices]

Fever duration (days): .

Maximum temperature of fever (℃) : .

Sore throat

Cough

Fatigue

Muscle soreness

Headache

Asymptomatic

Others: .

Hospitalization, medicine for COVID-19: .

Question 10: How many days of COVID-19 (nucleic acid or antigen) turn negative [fill in the blank]

Days: .

Question 11: How many days of COVID-19 symptoms last [fill in the blank]

Days: .

Question 12: Has the child been vaccinated with COVID-19 vaccine before the first infection [single choice]

Yes, number of inoculations: .

No

Question 13: Have the parents been vaccinated [single choice]

Yes, number of inoculations: .

No

Question 14: Current stage of the child's disease [single choice]

Intensive therapy phase

Maintenance phase

Drug withdrawal less than 1 year

Drug withdrawal for 1-3 years

Drug withdrawal for 3-5 years

Drug withdrawal for more than 5 years

Thank you for your support and cooperation!
